# Experimental study on mechanical and hydraulic properties of xanthan gum improved low liquid limit silty soil

**DOI:** 10.1038/s41598-024-61875-w

**Published:** 2024-05-14

**Authors:** Xutao Zhang, Wenyue Cao, Xiao Zhang

**Affiliations:** School of Architecture and Civil Engineering, Liaocheng University, Liaocheng, 252059 China

**Keywords:** Xanthan gum, Low liquid limit silty soil, Mechanical properties, Hydraulic properties, Improvement mechanism, Civil engineering, Composites

## Abstract

The low liquid limit silty soil in the North China plain area is generally unsuitable for direct use as roadbed and slope soil. In order to improve the performance of low liquid limit silty soil, xanthan gum was used as an improver. Through a series of tests, the improvement effect of xanthan gum on low liquid limit silty soil was studied. The test results showed that Xanthan gum as an improver could significantly improve the unconfined compressive strength of silty soil. With the increase in dosage and curing age, the unconfined compressive strength of improved silty soil continued to improve and eventually tended to stabilize. The optimal dosage and curing period were 2% and 7 days, respectively. In addition, Xanthan gum could greatly improve the permeability and disintegration of low liquid limit silty soil. The permeability coefficient of improved silty soil with a content of 0.75% Xanthan gum and a 7-day curing period was 4.73 × 10^−4^ m·s^−1^, which was only 1.10% of that of plain silty soil at the same curing period. After immersion in water for 12 h, the soil only experienced slight disintegration. The scanning electron microscope image showed that the gel generated by the hydration reaction of Xanthan gum could improve the compactness and integrity of the soil by filling the voids, thus significantly improving the mechanical and hydraulic properties of the low liquid limit silty soil.

## Introduction

In the Yellow River alluvial plain of China, there is a large amount of low liquid limit silty soil, with silt particle content generally reaching over 50% and clay particle content less than 10%. The special composition of soil particles determines its unique engineering performance. This type of silty soil has a low liquid limit, low strength, and extremely poor water stability, and is generally not suitable for use as a roadbed^1^. Therefore, the efficient low liquid limit silty soil improvement methods have always been an important challenge in engineering construction.

There are two main methods of soil improvement: physical and chemical improvement techniques. The physical improvement techniques are mainly achieved through replacement and compaction^2^. The soil replacement will waste a large amount of land resources, which does not meet the requirements of ecological and environmental protection. In addition, silty soil has low plasticity, poor cohesion and low strength, which makes it difficult to achieve the desired compaction level. At present, the chemical improvement techniques have been widely used in the field of geotechnical engineering. The cement, lime, asphalt, etc. are the most commonly used chemical improvement materials. However, the mass production and use of chemical improved materials have brought many environmental problems^3–5^. Therefore, it is necessary to use efficient and environmentally friendly materials to improve the soil.

The biopolymers are polysaccharides with a polymer chain structure. It is derived from natural resources and has a wide range of uses. Due to its rheological and pseudoplastic properties, it has been widely used as a thickener or emulsifier in food and medical products^6^. In the field of environmental engineering, the biopolymers are used for soil improvement^7^. In addition, the biopolymers are also used in the petroleum industry to guide the flow of oil^8^, as well as for crack repair in concrete^9^. The biopolymers have also received widespread attention in the field of geotechnical engineering due to their high efficiency in soil improvement and low environmental impact^10^. The research results indicate that^11–15^, guar gum, gellan gum, chitosan and carboxy methyl cellulose can effectively improve the strength of soil. Loose soil particles could be tightly bonded by Xanthan gum, therefore, the permeability of the improved soil was reduced, and the ability to resist hydraulic and wind erosion was enhanced^16–19^. In addition, the biopolymers are rich in carbon and nitrogen elements, which can provide nutrients for soil and promote plant growth. Therefore, the biopolymers can be used for slope vegetation protection and land desertification prevention^20–22^. Although the research results are encouraging, the biopolymers have not been widely used in the field of geotechnical engineering due to their market cost, on-site application conditions and methods, durability, and sensitivity to water^23^. Especially, xanthan gum can better adapt to different temperature and pH conditions^24^, and compared with other biopolymers, it has a larger production volume and relatively lower price^25^. As a green material, it has been used to improve various poor soils, such as high liquid limit coarse-grained soil^26^, clay^27–29^, sandy soil^30^, organic peat soil^31^ etc. The mechanical parameters such as unconfined compressive strength and shear strength of the improved soil have been significantly improved. Latifi et al.^32^ used Xanthan gum as an improver to treat cohesive soil, and the cohesive force of the improved soil was significantly increased. Chang et al.^33^ pointed out that the Xanthan gum has a better reinforcement effect on well graded soil, and the reinforcement effect mainly depends on four factors: soil type, xanthan gum content, curing age, and mixing method. The results of Ahmadi et al.^34–36^ showed that the cleaning improver such as xanthan gum can significantly improve the mechanical properties of soil after dry–wet cycle (W–D) and freeze–thaw cycle (F–T). Kumar et al.^24^ confirmed that the Xanthan gum is more efficient in reducing soil permeability compared to guar gum and pectin. Ghasemzade et al.^37^ conducted research on a new cleaning biopolymer. The results confirmed that Persian gum has successful properties in terms of bonding soil particles, pore filling, thermal stability, etc. Additionally, consuming smaller amounts of Persian gum in the presence of calcium chloride can make stabilization projects cost-effective^37,38^.

The Xanthan gum was selected as an improved material in this study, based on its wide applicability and low cost advantages. Currently, the research on xanthan gum-improved soils mainly focuses on sand and clay, with less attention paid to low liquid limit silty soil. In addition, most of the research has focused on the enhancement of the mechanical properties of soils, while less research has been conducted on the hydraulic properties, especially the collapse resistance, under geotechnical application conditions. The resistance of soils to disintegration is closely related to the protection of side slopes under rainfall conditions. The Xanthan gum was used to improve low liquid limit silty soil, and unconfined compressive strength tests, permeability tests, and wet disintegration tests were conducted. The influence of Xanthan gum on the mechanical and hydraulic properties of improved silty soil was studied, and its solidification mechanism was analyzed. The research results further enriches the method of roadbed improvement for low liquid limit silty soil. It provides an important theoretical basis for the application of xanthan gum in the engineering construction of such silty soil areas as well as the slope protection project.

## Test materials and methods

### Test materials

**Figure 1:**
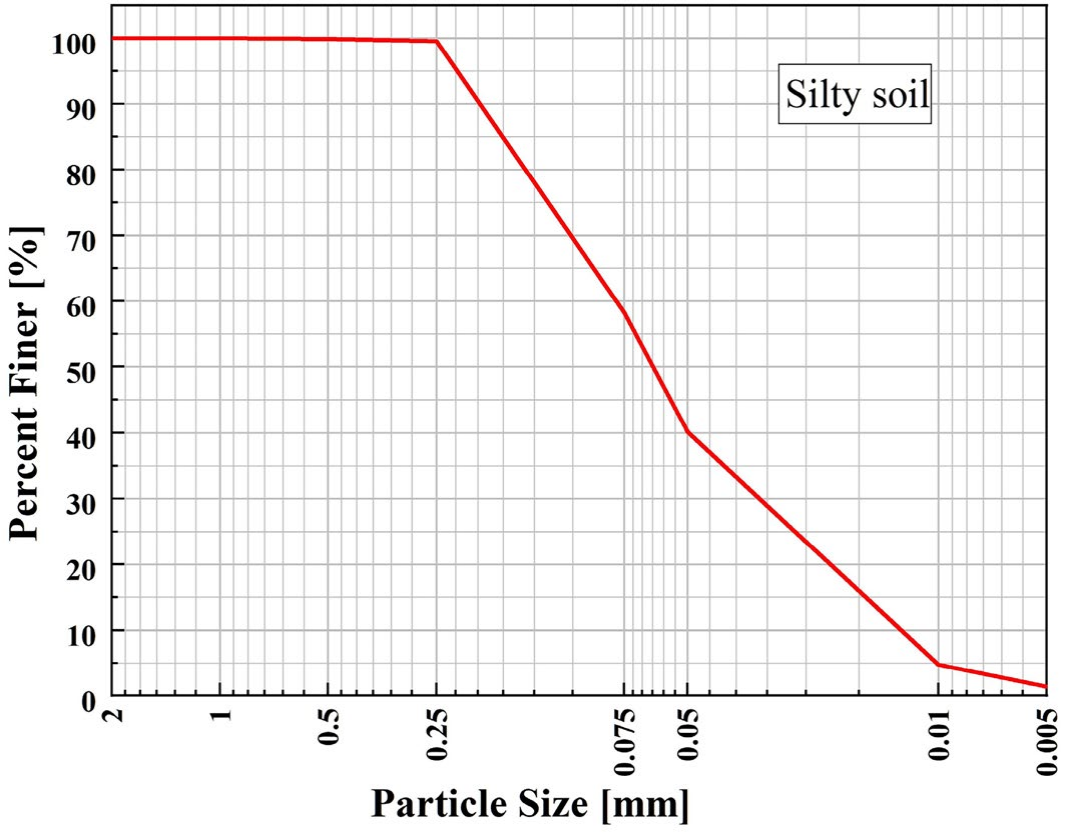
Particle grading curve of low liquid limit silty soil.

The basic physical indexes of the low liquid limit silty soil in this study are as follows: the liquid limit is 24.1%, the plastic limit is 15.4%, the plasticity index is 8.7, the optimum moisture content is 14.9%, the maximum dry density is 1.72 g/cm^3^, the specific gravity is 2.68, and the saturated moisture content is 40.45%. The content of soil particles with a grain size of less than 0.075 mm is 58.2%. The plasticity index of the soil is less than 10, the mass of particles larger than 0.075 mm does not exceed 50% of the total weight, and the liquid limit is less than 50. Therefore, the soil is defined as a low liquid limit silty soil. The content of soil particles with a particle size greater than 0.075 mm was less than 50%. Most of the soil particles were silty soil particles and a small number of sand particles and clay particles. When dry, the soil blocks are not firmly bonded enough, and can be rubbed by hand to form powder without any sticky feeling. When moist, it has fluidity and can be squeezed into a ball in the hand. The particle grading curve is shown in Fig. 1.

**Figure 2:**
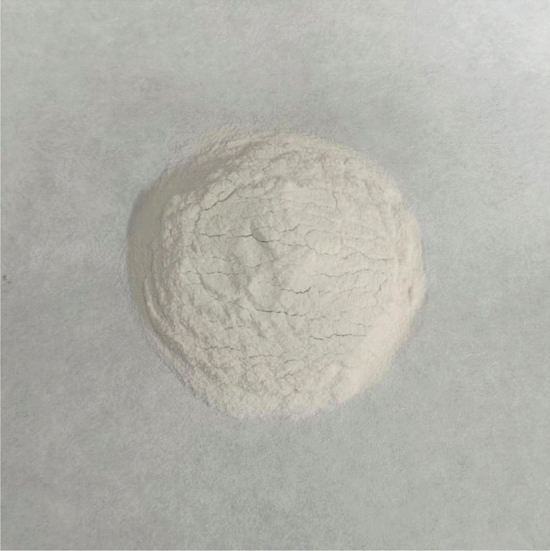
Xanthan gum.

The Xanthan gum is a polysaccharide produced by the fermentation of glucose or sucrose by *Xanthomonas* *flavus*^39^. It consists of two glucose, two mannose and one glucuronic acid unit. It is readily soluble in water, but insoluble in organic solvents such as ethanol, and has high viscosity rheological properties when dissolved in water. Xanthan gum used in this study was produced by Baikang Foods Factory and purchased from a retailer. It is 99.7% pure, light yellow powder (Fig. 2) and the pH value of 1% xanthan gum solution is 7.8. It should be sealed during storage to avoid moisture.

### Preparation of soil specimen

**Figure 3:**
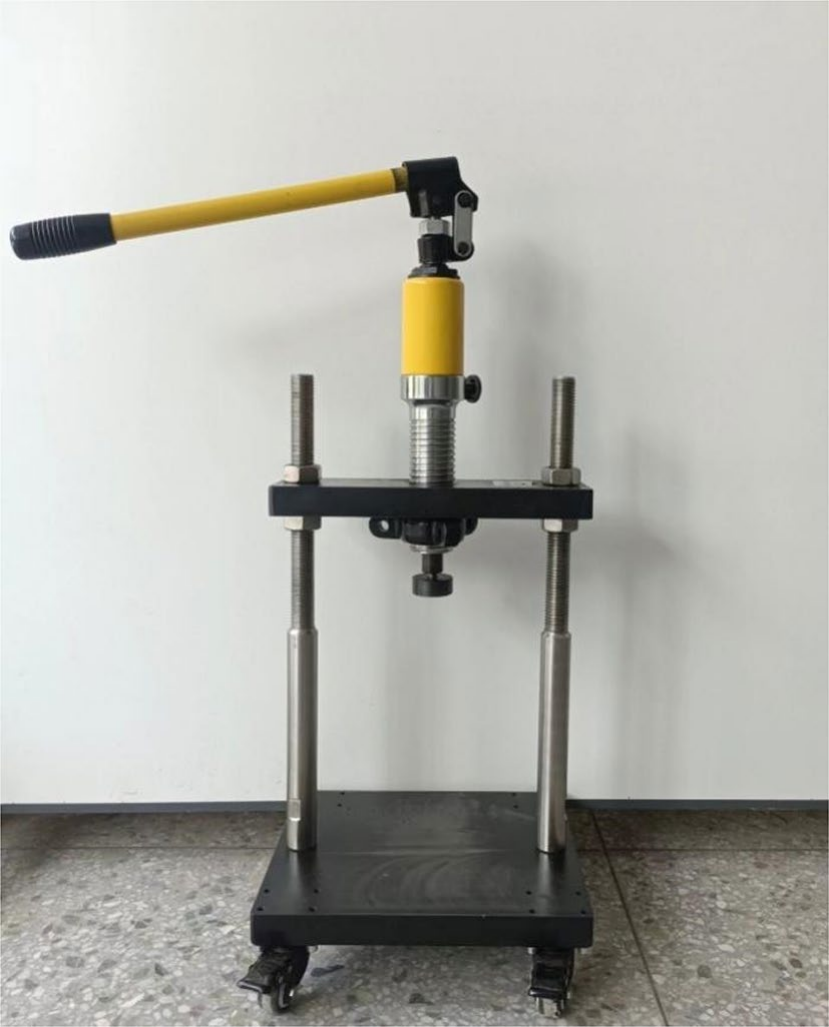
Soil specimens preparation instrument.

**Figure 4:**
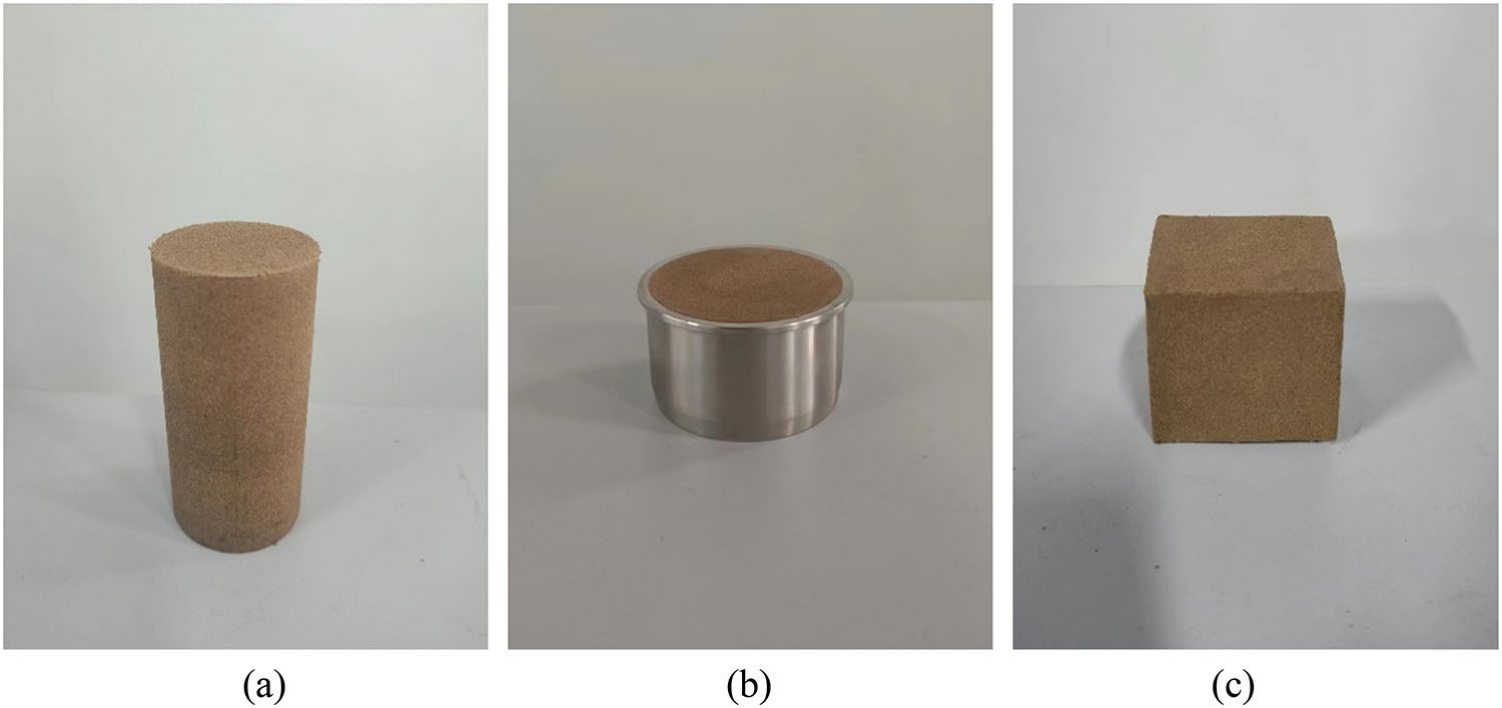
Soil specimens.

The dried and crushed silty soil is mixed evenly with a certain amount of Xanthan gum. Pure water is added to the mixed soil according to the optimal moisture content and stirred evenly. The mixed soil is sealed with plastic film. After 12 h, the water in the soil migrates uniformly, and then the soil specimen is prepared using the static pressure method. The soil specimens preparation instrument is shown in Fig. 3. To prepare the unconfined compressive specimen, the soil mixture was divided into three parts and poured sequentially into the mold for compaction. Then the specimen was pressed out with the soil extraction device for demolding. A standard cylindrical specimen with a height of 80 mm and a diameter of 39.1 mm was obtained as shown in Fig. 4a. The permeable soil specimen, which did not need to be demolded, was a 61.8 mm cylinder with a height of 40 mm, as shown in Fig. 4b. The disintegration test specimens were made into 95% compacted soil using a compaction device (same compaction as the above specimens), and amended to cubes with sides of 50 mm using a soil trimmer as shown in Fig. 4c. The soil body was avoided to be disturbed during the amendment process. The prepared earth samples were placed in a conservation box at a temperature of 25 ± 2 °C and 50% relative humidity and maintained to the appropriate age. Conduct the corresponding tests and record the test data according to <Standard for Soil Test Methods GBT 50123-201933>^36^.

### Test program

To investigate the effects of Xanthan gum content and curing age on the mechanical and hydraulic properties of the improved silty soil, the unconfined compressive strength test, permeability test, and wetting disintegration test were carried out. To analyze the solidification mechanism of Xanthan gum improved silty soil, the scanning electron microscopy experiments were carried out. The content of Xanthan gum and curing age of the soil specimens are detailed in Table 1.

**Table 1:** Test scheme.

Test items	Unconfined compressive strength test	Permeability test	Wetting disintegration test	SEM test
Xanthan gum content (%)	0.00			
	0.50	0	0	
	1.00	0.25	0.25	0
	1.50	0.5	0.5	3
	2.00	0.75	0.75	
	2.50			
	3.00			
Curing age (d)	1	1		
	4	4		
	7	7	7	7
	14	28		
	28			

### Unconfined compressive strength test

The unconfined compressive strength test was carried out by the electronic universal testing machine. The maximum range of the testing machine was 10 kN. The test was carried out with equal strain loading at a loading rate of 1 mm/min. When the pressure reached the peak, continued to add 3–5% strain until the soil sample was completely destroyed. During the experiment, the pressure and deformation of the soil specimens were tested. The data were averaged and analysed to reduce the error. The specimens with excessive errors were excluded to ensure that compressive strengths of the three parallel specimens did not exceed 10% of their mean values.

### Permeability test

**Figure 5:**
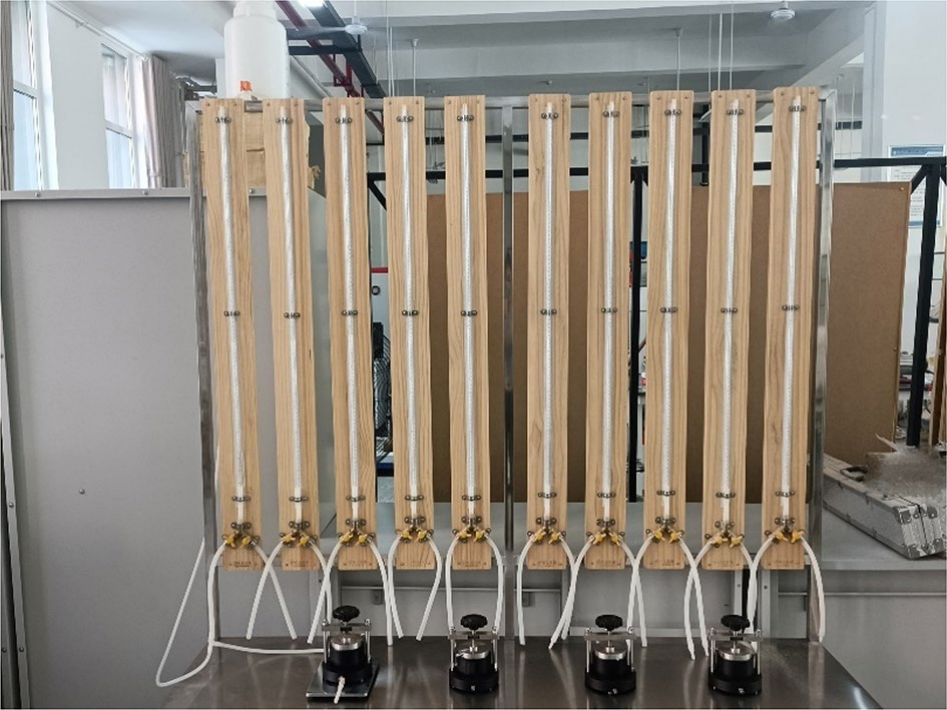
WST-2 Falling Head Permeameter.

In geotechnical engineering, seepage can cause deformation of soil and have adverse effects on the stability of geotechnical structures. The influence of Xanthan gum content and curing age on the permeability performance of improved silty soil was studied using the WST-2 Falling Head Permeameter (Fig. 5). The test procedure are as follows:

1. Soak the silty soil specimens in water to saturation.
2. Apply Vaseline to the inner wall of the sleeve, install the saturated soil specimen into the sleeve, press in the waterproof gasket, and install the permeable stone. Then tighten the upper and lower covers to ensure no air or water leakage.
3. Connect the infiltration container with the water head device, inject water into the infiltration container, remove the air from the container. The test began after the water from the outlet pipe was stable.
4. During the test, the head was filled to a certain height, and the starting head h_1_ and the starting time t_1_ were measured. After an interval of time t, the head h_2_ was measured. The starting and ending water temperatures were recorded. Repeat the test 5 times for each specimen. Finally, averages were taken and analyzed, and it was ensured that each test result was within 10% of the average.

### Wetting disintegration test

**Figure 6:**
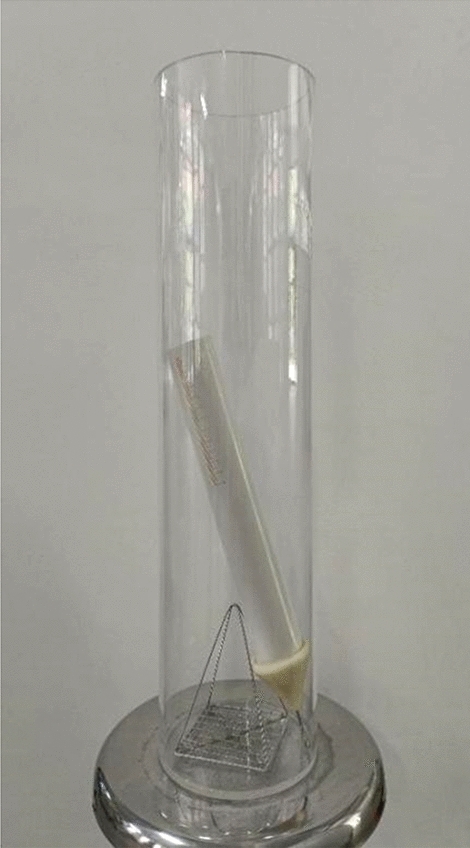
Wetting disintegration instrument.

The disintegration of soil is caused by the infiltration of water, which weakens the bonding force between soil particles and leads to the disintegration of the soil. The disintegration rate can be used as an indicator to determine the water stability of soil. The wetting disintegration instrument consists of a water container, a hanging net, and a float bowl, as shown in Fig. 6. During the test, the silty soil specimen is placed on a hanging net and then placed in a water container. After a period of time, the reading of the float bowl is recorded. The disintegration rate is calculated using the following formula:

1 $${N}_{t}=\frac{({m}_{t}-{m}_{a})}{({m}_{b}-{m}_{a})} \cdot 100\%$$

where *N*_t_—disintegration rate of soil specimen at *t* time (When *N*_t_ > 0, the soil specimen exhibits mainly water absorption. When *N*_t_ < 0, the soil specimen exhibits mainly disintegration); *m*_*t*_—reading of float bowl at time *t*; *m*_*a*_—reading of float bowl placing soil specimen; *m*_*b*_—reading of float bowl without placing soil specimen.

### Scanning electron microscopy test

To study the microstructure of Xanthan gum improved silty soil and analyze its solidification mechanism, the scanning electron microscopy tests were carried out. The specimens of plain silty soil and improved silty soil with a content of 3% Xanthan gum were analyzed. The specimen was cured for 7 days in an environment with a temperature of 25 ± 2 °C and a relative humidity of 50%. Before the experiment, the soil was fully ground and evenly spread on the slides to ensure that the surface of the sample was flat. Subsequently, the SEM specimens (about 1 cm^2^) were placed in an ion sputtering apparatus for gold spraying to make the surface conduct electricity. During the test, the microstructure of the soil was observed using a Quanta 600FEG scanning electron microscope. The magnification and focal length were adjusted, and the image was photographed when it was clear. About 5 images were taken for each type of gel-soil interaction.

## Unconfined compressive strength test results and analysis

The unconfined compressive strength test of silty soil specimens was carried out using an electronic universal testing machine, and the pressure and deformation of the specimens were recorded during the test process. The unconfined compressive strength can be obtained by dividing the maximum pressure of the soil specimen by its cross-sectional area. Based on test data, the complete stress–strain curve of the soil specimens can be obtained. The unconfined compressive strength of silty soil specimens under different contents of Xanthan gum and curing age conditions is shown in Table 2.

**Table 2:** The unconfined compressive strength of silty soil specimens (kPa).

Xanthan gum content (%)	Curing age (d)				
	1	4	7	14	28
0.00	232.83	764.66	916.26	1168.36	1273.05
0.25	369.35	1528.50	2594.68	2670.06	2931.37
0.50	544.39	2086.29	3246.28	3739.59	3894.53
1.00	650.76	2751.3	4339.26	4826.71	4856.02
1.50	764.66	3547.79	4699.40	5046.98	5351.01
2.00	864.33	4129.04	5350.66	5528.56	5777.31
2.50	915.42	4402.08	5518.51	5631.58	5796.57
3.00	956.46	4649.15	5717.01	5864.41	6077.15

### Effect of xanthan gum content and curing ages on unconfined compressive strength

**Figure 7:**
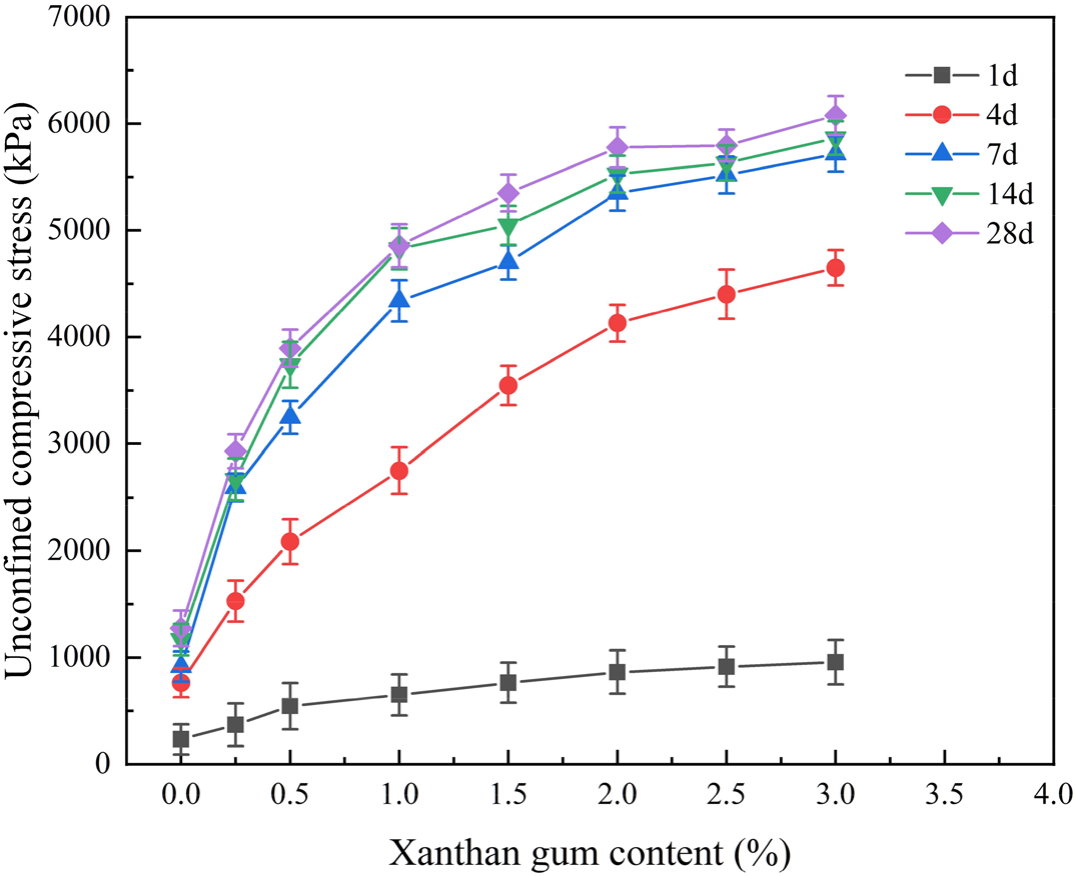
Curves of unconfined compressive strength with Xanthan gum content under different curing ages.

Based on the test data in Table 2, the curves of unconfined compressive strength with Xanthan gum content under different curing ages were plotted, as shown in Fig. 7. As shown in Fig. 7, with the increase of Xanthan gum content, the unconfined compressive strength of the improved silty soil continuously increases. When the content of Xanthan gum is 0.25% and the curing age is 28 days, the unconfined compressive strength of the improved silty soil is 2931.37 kPa. Compared to the same curing age of plain silty soil, the unconfined compressive strength has increased by 2.30 times. This indicates that for low liquid limit silty soil, adding a small content of Xanthan gum can also achieve good improvement effects. When the Xanthan gum content is 3% and the curing age is 28 days, the unconfined compressive strength of the improved silty soil reaches the highest value of 6077.15 kPa. It is 4.77 times that of plain silty soil with the same curing age. In addition, when the Xanthan gum content exceeds 2%, the unconfined compressive strength of the improved silty soil tends to be stable. For example, when the curing age is 7 days, the unconfined compressive strength of plain silty soil is 916.26 kPa. The unconfined compressive strength of improved silty soil with 0.25%, 0.5%, 1%, 1.5%, 2%, 2.5%, and 3% Xanthan gum is 2594.68, 3246.28, 4339.26, 4699.40, 5350.66, 5518.51 and 5717.01 kPa, respectively. Compared with the plain silty soil, the compressive strength is increased by 183.18%, 254.29%, 373.58%, 412.88%, 483.96%, 502.28%, and 523.95%, respectively. It can be seen that the most economical and efficient Xanthan gum content is 2%.

**Figure 8:**
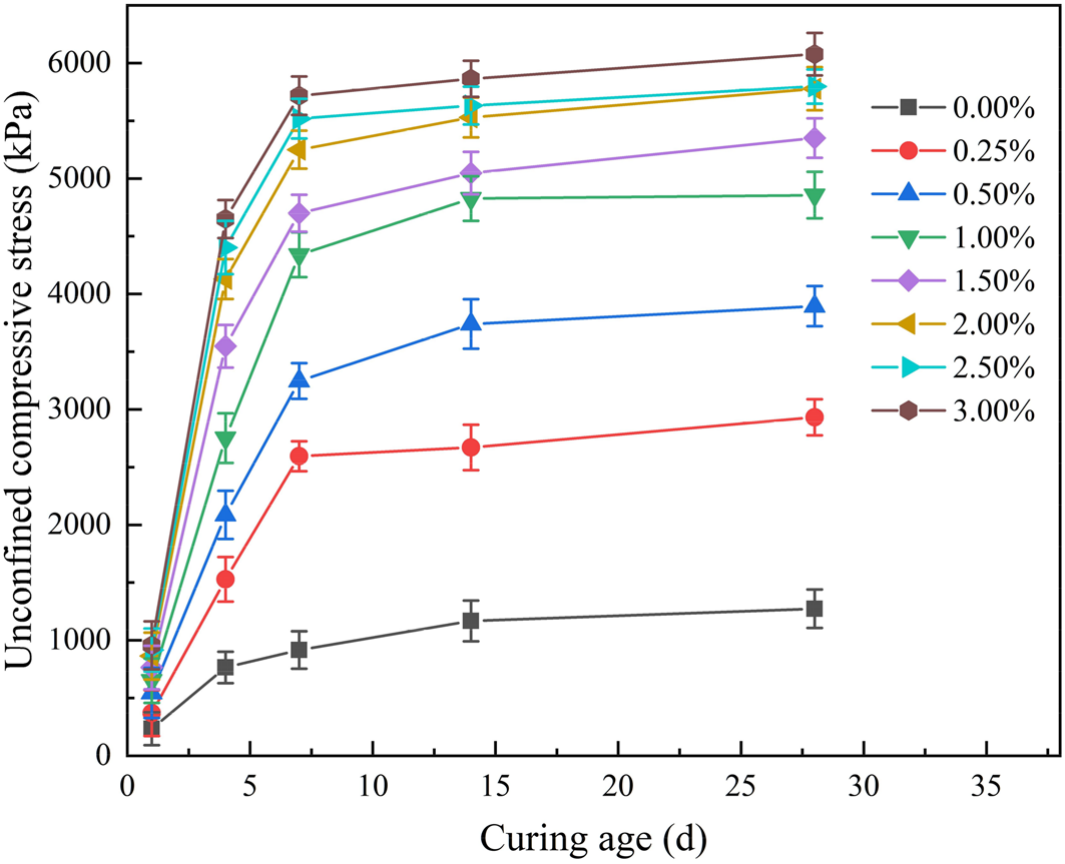
Curves of unconfined compressive strength with curing ages under different Xanthan gum content.

Based on the data in Table 2, the curves of unconfined compressive strength with curing age under different Xanthan gum content were plotted, as shown in Fig. 8. Under the same Xanthan gum content, the unconfined compressive strength of improved silty soil increases with the increase of curing age. The compressive strength of the soil increases rapidly during the 7 days curing age. After 7 days, the compressive strength tends to be stable. For example, when the curing age is 7 days, the unconfined compressive strength of 0.5% Xanthan gum improved silty soil is 4649.99 kPa, which is 90.3% of the strength at 28 curing days. The compressive strength of plain silty soil also increases slightly with the increase of curing age due to the increase of its cohesion. However, after curing, the unconfined compressive strength of plain silty soil is still significantly lower than that of the improved silty soil. It can be obtained that the most economical and efficient curing age for Xanthan gum improved silty soil is 7 days. This not only ensures that the strength of the silty soil is increased but also helps to shorten the construction period. This conclusion provides good engineering guidance for the engineering application of Xanthan gum to improve silty soil.

### Complete stress–strain curve of silty soil

**Figure 9:**
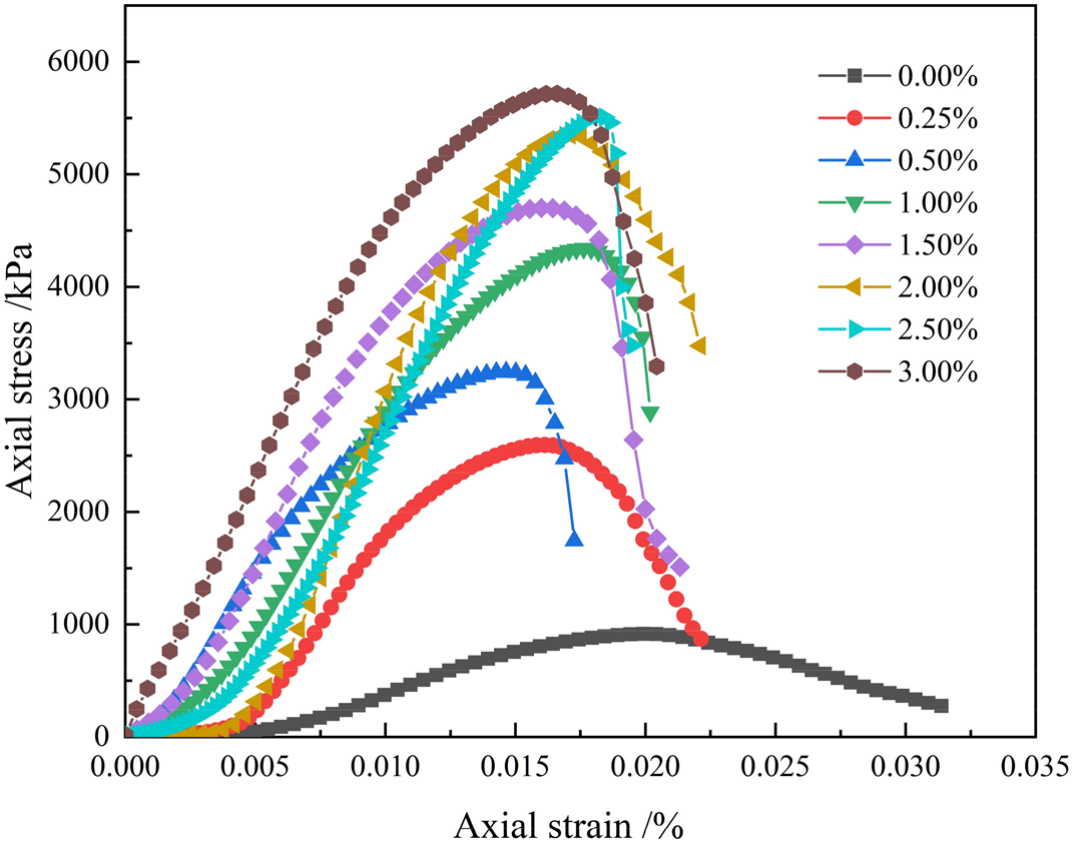
Complete stress–strain curves of silty soil.

The complete stress–strain curves of improved silty soil with different Xanthan gum content at 7 days curing age are shown in Fig. 9. The curves can be divided into three stages: (1) Initial compaction stage: The stress–strain curve shows an upward concave shape, and the tangent modulus is relatively small. The soil specimen is mainly compacted. (2) Stress strengthening stage: The stress–strain curve is approximately a straight line, and the tangent modulus increases. The stress increases rapidly with the increase of strain. (3) Failure stage: The stress of soil specimen rapidly decreases after reaching its peak, indicating that soil specimen has lost its bearing capacity. The peak stress is the unconfined compressive strength of soil specimen.

As shown in Fig. 9, the peak stress of the improved silty soil continuously increases with the increase of Xanthan gum content. The peak strain of the soil specimen varies between 1.40% and 2.00%. The peak strain of plain silty soil is 1.99%. Compared with plain silty soil, the peak strain of improved silty soil generally decreases with the increase of Xanthan gum content. In the failure stage, the stress of plain silty soil decreases relatively slowly after reaching its peak stress, while the stress of improved silty soil decreases rapidly. The Xanthan gum can improve the unconfined compressive strength of low liquid limit silty soil, but it weakens the plastic deformation ability of the silty soil, making it prone to brittle failure.

### Failure mode of silty soil specimens

**Figure 10:**
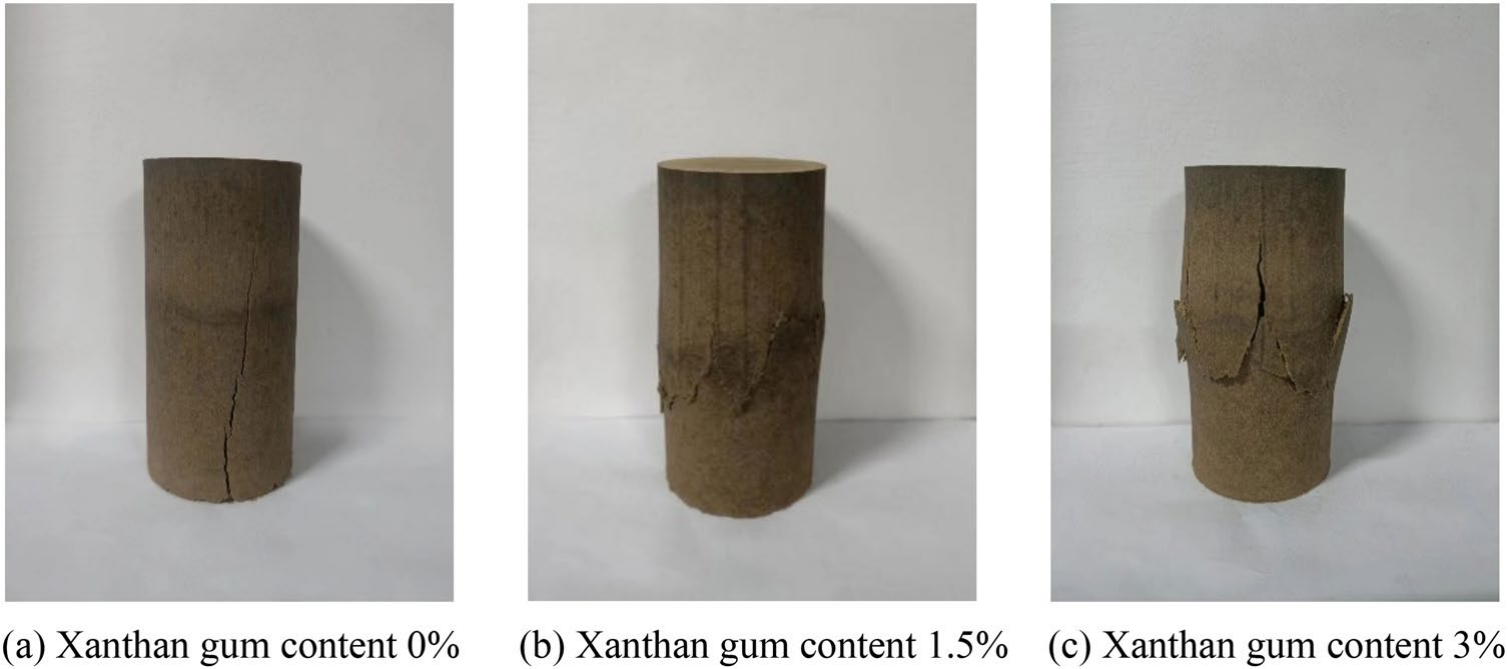
Failure mode of silty soil specimens.

The failure modes of the plain silty soil and the improved silty soil with 1.5%, 3% Xanthan gum content are shown in Fig. 10. As shown in Fig. 10a, the destroyed plain silty soil specimen has obvious shear bands. The inclination angle of the shear band is approximately 75°. The damage cracks are relatively complete and have a good connection. The specimen surface is relatively smooth, and there is no significant change in its diameter. As shown in Fig. 10b,c, the destroyed Xanthan gum improved silty soil specimen has a certain lateral dilation. The soil clods are warped along the shear strip. There are many small cracks on the surface of the specimen. The reason for the above phenomenon is that the gel formed by xanthan gluing reaction enhances the adhesion between soil particles, and cements the originally loose silty soil particles. The soil particle skeleton was strengthened, and the soil integrity was enhanced. Therefore, when the unconfined compressive strength test is carried out, the failure of the soil is delayed, and the soil has a better ability to resist deformation. Finally, the compressive strength of soil is improved.

## Permeability test results and analysis

**Figure 11:**
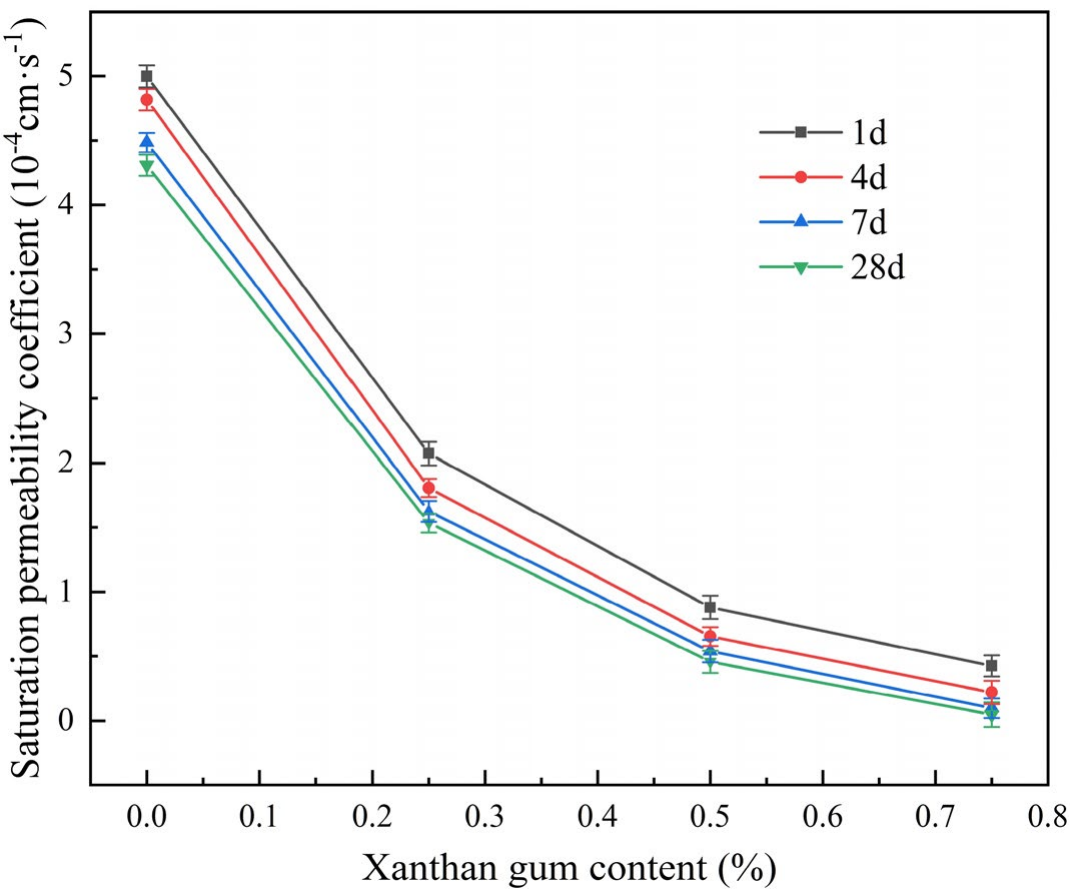
Curves of permeability coefficient with Xanthan gum content under different curing ages.

The permeability test was carried out by WST-2 permeameter in the form of the falling head. The head data and test interval were recorded. The permeability coefficient of the soil specimen was calculated by using Eq. (1). The permeability coefficient of soil specimens under different Xanthan gum content and curing ages was shown in Table 3. Based on the permeability coefficient data, the curves of the permeability coefficient with Xanthan gum content under different curing ages were plotted, as shown in Fig. 11.

**Table 3:** Permeability coefficient of silty soil specimens (10^−4^ cm/s).

Xanthan gum content (%)	Curing age (d)			
	1	4	7	28
0.00	4.99	4.81	4.48	4.30
0.25	2.07	1.80	1.62	1.53
0.50	0.87	0.65	0.53	0.45
0.75	0.42	0.21	0.09	0.04

As can be seen in Fig. 11, the permeability coefficient of the soil specimen decreases with the increase of Xanthan gum content and curing age, and the soil permeability coefficient is more significantly affected by the Xanthan gum content. The permeability coefficient of improved silty soil is significantly reduced due to the addition of 0.25% Xanthan gum content. When the Xanthan gum content is 0.75% and the curing age is 28 days, the permeability coefficient of the improved silty soil has decreased to 4.73 × 10^−6^ cm·s^−1^, which is only 1.10% of the plain silty soil in the same curing age. Relatively, the permeability of improved silty soil is not significantly affected by the curing age. When the curing age is more than 7 days, the permeability coefficient tends to be stable. The reason for the above-mentioned experimental phenomenon is: The permeability of soil is mainly affected by its porosity. The gel formed by the Xanthan gum hydration reaction can effectively fill the void of the soil. Therefore, the seepage path of the water is blocked^40^. Furthermore, the gel contains a large number of highly reactive hydrophilic groups to adsorb water molecules, which also slows down the seepage of water. With the increase of curing age, the hydration reaction of Xanthan gum is more sufficient, the cementation effect between the gel and the soil particles is stronger. As a result, the permeability coefficient of the soil becomes smaller.

## Wetting disintegration test

### Comparative analysis of soil specimens disintegration phenomenon

**Figure 12:**
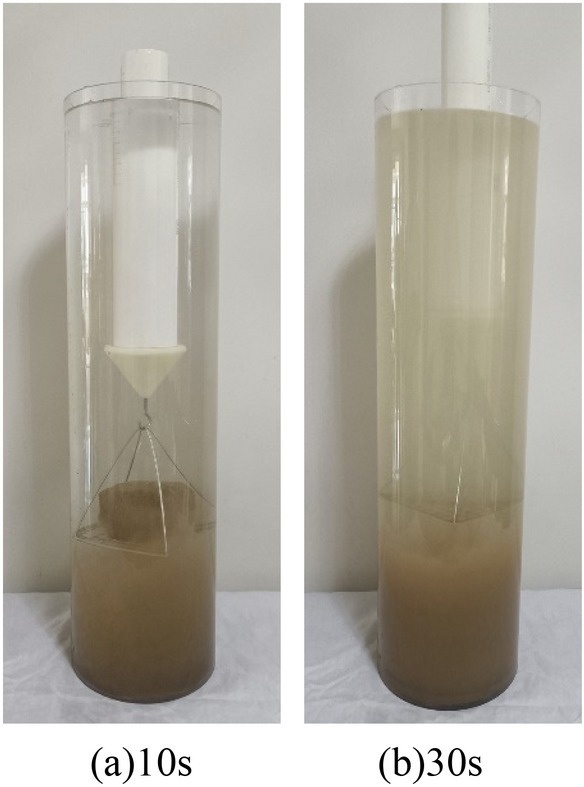
Disintegration phenomenon of plain silty soil.

**Figure 13:**
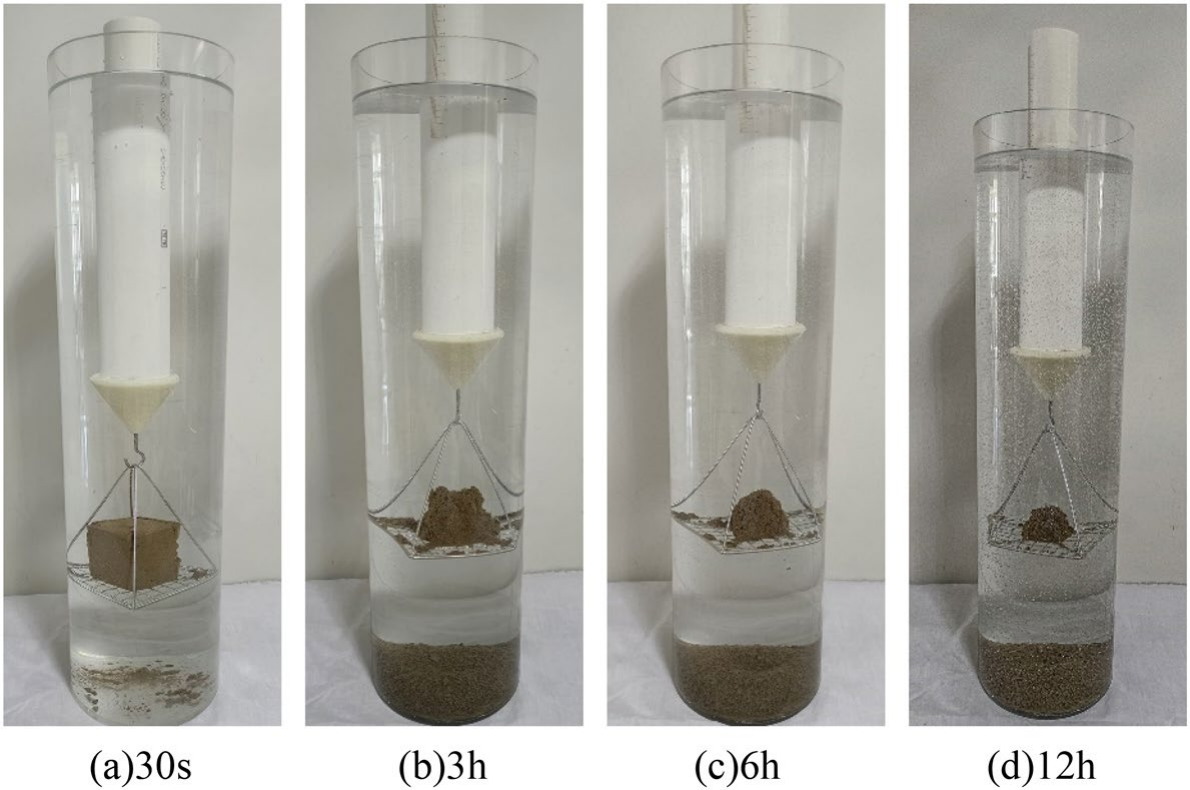
Disintegration phenomenon of improved silty soil with 0.25% Xanthan gum.

**Figure 14:**
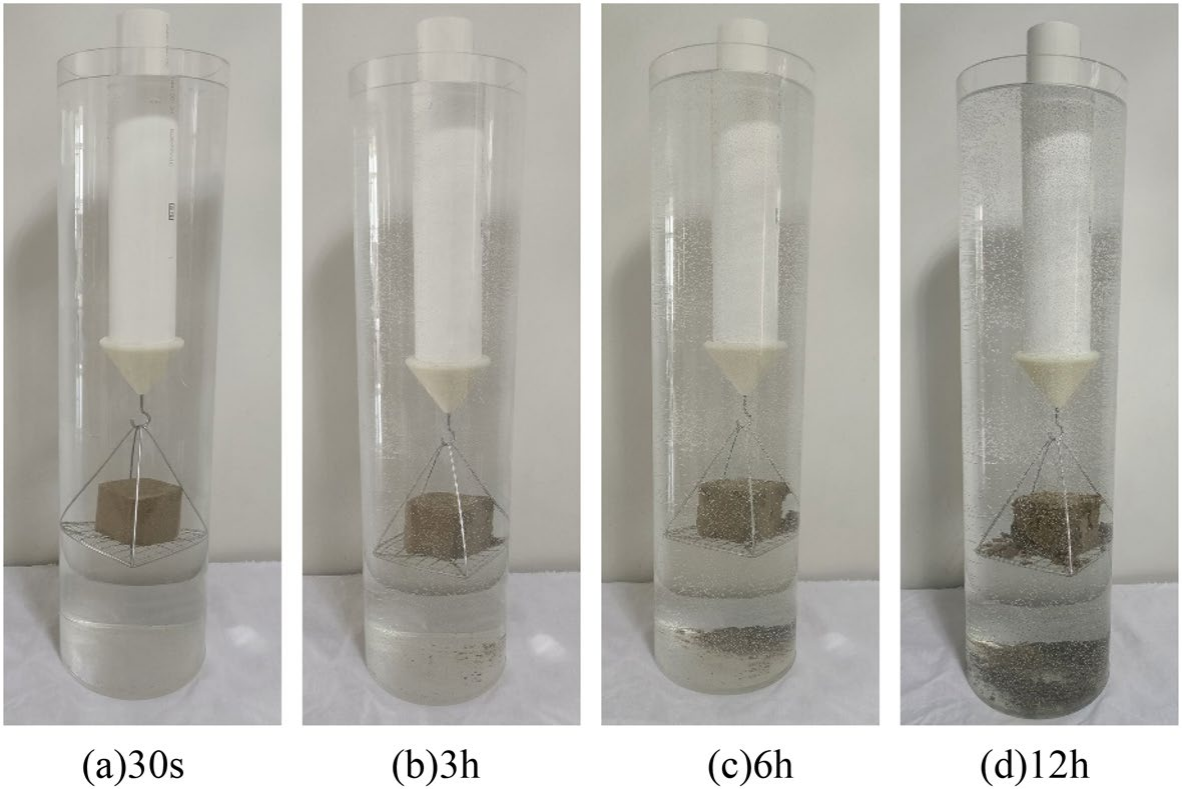
Disintegration phenomenon of improved silty soil with 0.50% Xanthan gum.

**Figure 15:**
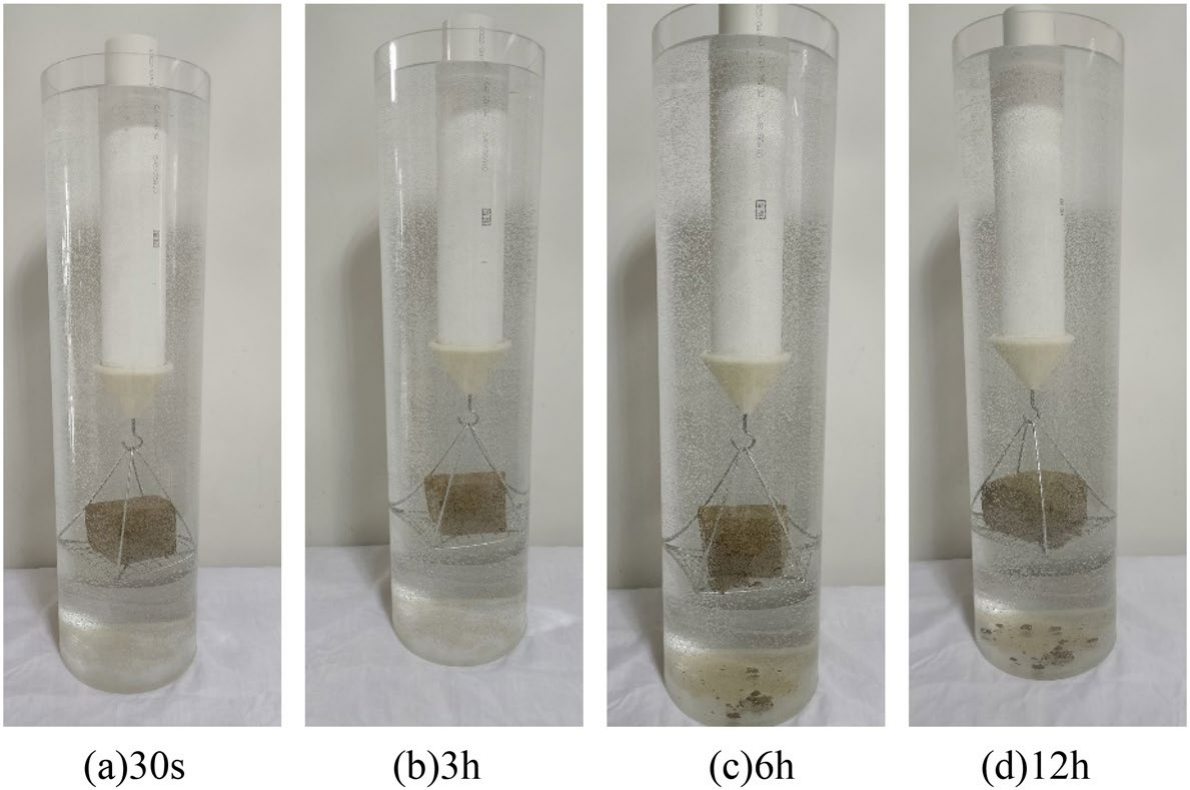
Disintegration phenomenon of improved silty soil with 0.75% Xanthan gum.

The wetting disintegration tests were carried out using plain silty soil specimens and improved silty soil specimens with 0.25%, 0.50%, and 0.75% Xanthan gum, and the curing age of the soil specimens was 7 days. The disintegration process of soil specimens includes four stages: wetting, softening, collapsing, and stable disintegration. The disintegration phenomenon of different soil specimens is as follows: (1) Although the plain silty soil specimen has been reshaped and compacted, its water stability was still very poor. After immersion, the water immediately became turbid. The soil specimen completely disintegrates within 30 s, as shown in Fig. 12. (2) The water stability of the improved silty soil with 0.25% Xanthan gum was significantly improved. After 30 s of immersion in water, the cracks appeared on the surface of the specimen, and slight spalling occurred. After immersion for 3 h, the specimen partially collapsed. After immersion for 6 h, most of the specimen disintegrated. After immersion for 12 h, there were more bubbles, but the specimen did not completely disintegrate, as shown in Fig. 13. (3) The water stability of improved silty soil with a content of 0.5% Xanthan gum is relatively good. After immersion for 3 h, the cracks and spalling occurred in the specimen. After immersion for 6 h, the number of bubbles increased and the specimen began to peel off. After immersion for 12 h, a small part of the specimen disintegrated with a large number of bubbles, as shown in Fig. 14. (4) The water stability of improved silty soil with a content of 0.75% Xanthan gum is excellent. After immersion in water, a large number of bubbles are generated, but no peeling occurs. After immersion for 3 h, a slight peeling phenomenon occurred. After 12 h of immersion, the specimen was still stable with a small amount of disintegration, as shown in Fig. 15.

### Comparative analysis of soil specimens disintegration rates

**Figure 16:**
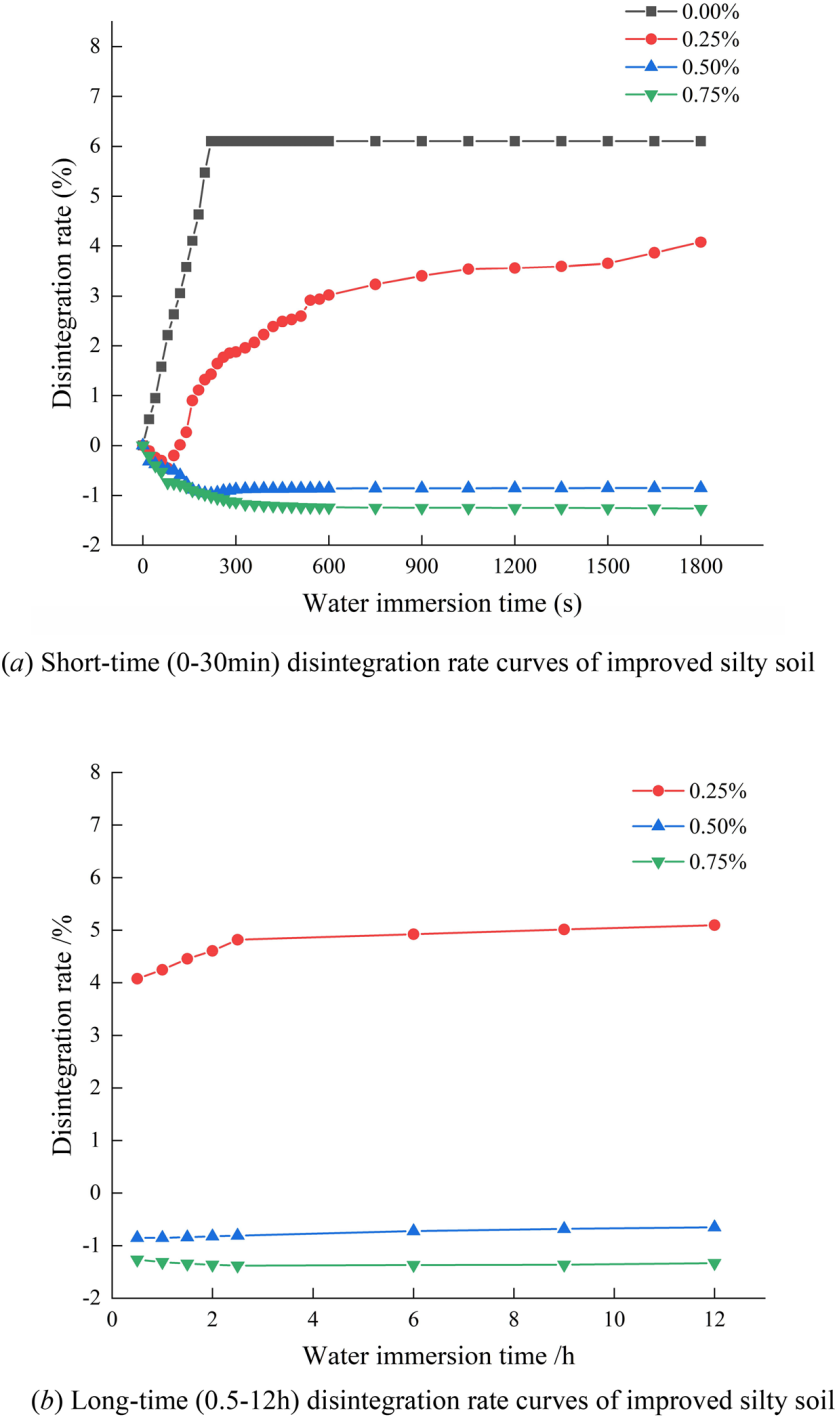
Disintegration rate curves of improved silty soil.

The reading of float bowl was recorded during the wetting disintegration test. The disintegration rate of the soil specimen at time *t* can be calculated using formula (1). To demonstrate the variation pattern of disintegration rate over time more clearly, the variation curve of disintegration rate is drawn in two sections. That is, the short-term (0–30 min) disintegration rate curve and the long-term (0.5–12 h) disintegration rate curve, as shown in Fig. 16.

Based on Fig. 16a, the following conclusions can be drawn: (1) The disintegration rate of the plain silty soil specimen increases rapidly over time, and the specimen rapidly peels off and disintegrates after being immersed in water. After immersion in water for 3 min, the specimen basically disintegrated. The reason for the above phenomenon is that due to the loss of cementitious materials between particles, the structure of the plain silty soil is rapidly destroyed, and the stability is lost. (2) The disintegration rate of the improved silty soil with 0.25% Xanthan gum shows a pattern of first decreasing and then increasing rapidly. At first, the disintegration rate of the improved silty soil was negative because the soil structure could not be rapidly destroyed after immersion. The soil specimen continuously absorbs water and expands, resulting in an increase in its weight. In the later stage, the improved silty soil specimen showed significant disintegration due to the wetting effect, and the disintegration rate transformed into a positive increase. After immersion for 30 min, the disintegration rate of the soil specimen reached 31.17%. (3) The improved silty soil with a content of 0.50% Xanthan gum has similar disintegration characteristics as the improved silty soil with a content of 0.25%, but its disintegration rate significantly decreases. Within 30 min of immersion in water, the soil specimen only showed slight disintegration and overall exhibited water absorption. (4) The improved silty soil with 0.75% Xanthan gum does not peel and disintegrate within 30 min. The disintegration rate has been showing a negative increase due to continuous water absorption.

The disintegration rate change curve of improved silty soil over a long period of time (0.5–12 h) reflects the influence of Xanthan gum content on the long-term water stability, as shown in Fig. 16b. (1) The disintegration rate of the improved silty soil with 0.25% Xanthan gum increases rapidly within 1 h. After 1 h, the disintegration rate gradually slows down. After 12 h, the disintegration rate was 80.20%. (2) The improved silty soil with 0.50% Xanthan gum has better water stability. After 12 h, the disintegration rate was − 10.17%, which is due to the weight of the soil specimen absorbing water being greater than the weight of peeling. (3) The improved silty soil with 0.75% Xanthan gum has the best water stability. After 12 h, the disintegration rate was − 20.98%, and the soil specimen hardly experienced disintegration.

In summary, the Xanthan gum can significantly improve the water stability of low liquid limit silty soil, and the Xanthan gum content has a significant impact on the water stability of improved silty soil. The water stability of improved silty soil gradually improves with the increase of Xanthan gum content. When the content of Xanthan gum is 0.25%, the water stability of the improved silty soil is still poor. When the content of Xanthan gum is 0.50%, the water stability is better. When the content of Xanthan gum is 0.75%, the water stability is the best.

## Analysis of improvement mechanism of improved silty soil

**Figure 17:**
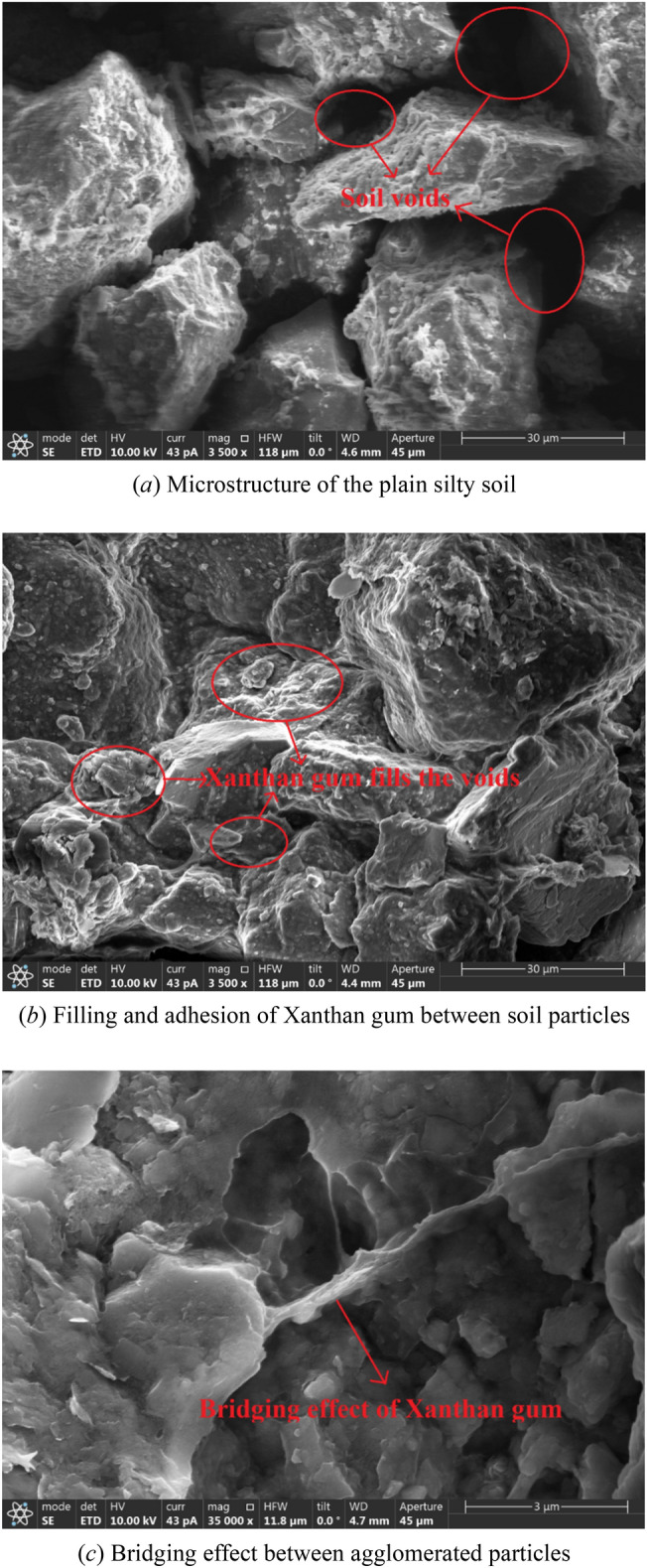
Microstructure of silty soil specimens.

Using the Quanta 600FEG field emission scanning electron microscopy, the microstructure of improved silty soil with a content of 3% Xanthan gum and plain silty soil was observed, and the SEM images were obtained, as shown in Fig. 17.

According to Fig. 17a, it can be seen that the pores between the particles of plain silty soil are large, with a high porosity, and the soil particles have clear edges and corners. The connection between soil particles is mainly through point contact, while the soil is mainly composed of overhead structures between particles, which is the reason for the lower strength of plain silty soil. The gel formed by the hydration of Xanthan gum can fill the gaps between soil particles and attach to the surface of soil particles, which makes the soil particles show agglomeration and bonding effect, making a single soil particle form a larger aggregate, as shown in Fig. 17b. There is a bridging connection between the agglomerated particles, which enables the soil particles in the improved soil to form a stable structure, as shown in Fig. 17c. Therefore, the effects of Xanthan gum on improving silty soil can be summarized into three types: filling bonding, wrapping bonding, and bridging bonding.

The improvement mechanism of Xanthan gum on low liquid limit silty soil can be explained as follows: First, the soil void was effectively filled. The original large voids of the soil were filled by a gel formed by the hydration of Xanthan gum, which not only improved the compactness of the soil but also blocked the water leakage path. As a result, the permeability and water stability of the soil were significantly improved. Second, the loose silty soil particles were cemented by gel, which limited the movement of soil particles, strengthened the soil particle skeleton, enhanced the integrity of the soil, and significantly improved the strength of the soil. In summary, the mechanical and hydraulic properties of low limit silty soil were significantly improved by adding a small amount of Xanthan gum.

## Conclusion

The biopolymer Xanthan gum was used as an improver to treat low liquid limit silty soil, and the unconfined compressive test, permeability test, and wetting disintegration test were carried out to study the effect of Xanthan gum on the mechanical and hydraulic properties of low liquid limit silty soil. The improvement mechanism of Xanthan gum on silty soil has been studied. The following conclusions were drawn:

1. The unconfined compressive strength of Xanthan gum improved low liquid limit silty soil increased rapidly during the 7-day curing age, after which the growth rate slowed down and gradually stabilized. The unconfined compressive strength of the improved silty soil increased continuously with the increase of Xanthan gum content. However, when the content of Xanthan gum was increased to 2%, the increase in strength of the improved silty soil was slowed down. The experimental study showed that the optimum content and the optimum curing age of Xanthan gum improved silty soil were 2% and 7 days, respectively.
2. The Xanthan gum could effectively improve the permeability of low liquid limit silty soil. The permeability coefficient of improved silty soil decreased with the increase of Xanthan gum content and the curing age. When the dosage of Xanthan gum was very small (such as 0.25%), the permeability of improved silty soil could be significantly improved. Compared with plain silty soil, the permeability coefficient of improved silty soil with a content of 0.75% Xanthan gum and a curing age of 7 days decreased by 98.90%.
3. After immersion, the low liquid limit silty soil rapidly disintegrated. By adding a small amount of Xanthan gum, the water stability of the silty soil could be significantly improved. When the dosage of Xanthan gum was 0.75%, there was almost no disintegration within 12 h, and its disintegration rate was − 20.98%.
4. The scanning electron microscope image showed that the gel generated by the hydration reaction of Xanthan gum could improve the compactness and integrity of the soil by filling the voids and cementing silty soil particles. Thus the mechanical and hydraulic properties of the low limit silty soil had been significantly improved.
5. Xanthan gum is a kind of biopolymer with the advantages of being neutral, environmentally friendly and renewable. The mechanical and hydraulic properties of the improved silty soil were significantly improved, which proved the high efficiency of xanthan gum in improving the properties of silty soil. The results provide an important reference for the dosage of xanthan gum and the selection of maintenance time in the improvement process. It has great value in the application of roadbed improvement, slope rainfall protection and engineering construction in the area of low liquid limit silty soil.

## Data Availability

All data generated or analysed during this study are included in this published article or are available from the corresponding author on reasonable request.
